# Predicting compressive strength of eco-friendly plastic sand paver blocks using gene expression and artificial intelligence programming

**DOI:** 10.1038/s41598-023-39349-2

**Published:** 2023-07-27

**Authors:** Bawar Iftikhar, Sophia C. Alih, Mohammadreza Vafaei, Muhammad Faisal Javed, Muhammad Faisal Rehman, Sherzod Shukhratovich Abdullaev, Nissren Tamam, M. Ijaz Khan, Ahmed M. Hassan

**Affiliations:** 1 School of Civil Engineering, Universiti Teknologi Malaysia, 81310 Johor Bahru, Johor Malaysia; 2 Department of Civil Engineering, COMSATS University Islamabad, Abbottabad Campus, Abbottabad, 22060 Pakistan; 3 Institute of Noise and Vibration, School of Civil Engineering, Universiti Teknologi Malaysia, 81310 Johor Bahru, Johor Malaysia; 4 Department of Architecture, University of Engineering and Technology Peshawar, Abbottabad Campus, Abbottabad, Pakistan; 5 Faculty of Chemical Engineering, New Uzbekistan University, Tashkent, Uzbekistan; 6 Department of Science and Innovation, Tashkent State Pedagogical University Named after Nizami, Bunyodkor Street 27, Tashkent, Uzbekistan; 7 Department of Physics, College of Science, Princess Nourah Bint Abdulrahman University, P.O. Box 84428, 11671 Riyadh, Saudi Arabia; 8 Department of Mathematics and Statistics, Riphah International University, I-14, Islamabad, 44000 Pakistan; 9 Department of Mechanical Engineering, Lebanese American University, Kraytem, Beirut, 1102-2801 Lebanon; 10 Center of Research, Faculty of Engineering, Future University in Egypt, New Cairo, 11835 Egypt

**Keywords:** Climate sciences, Environmental sciences

## Abstract

Plastic sand paver blocks provide a sustainable alternative by using plastic waste and reducing the need for cement. This innovative approach leads to a more sustainable construction sector by promoting environmental preservation. No model or Equation has been devised that can predict the compressive strength of these blocks. This study utilized gene expression programming (GEP) and multi-expression programming (MEP) to develop empirical models to forecast the compressive strength of plastic sand paver blocks (PSPB) comprised of plastic, sand, and fibre in an effort to advance the field. The database contains 135 results for compressive strength with seven input parameters. The R^2^ values of 0.87 for GEP and 0.91 for MEP for compressive strength reveal a relatively significant relationship between predicted and actual values. MEP outperformed GEP by displaying a higher R^2^ and lower values for statistical evaluations. In addition, a sensitivity analysis was conducted, which revealed that the sand grain size and percentage of fibres play an essential part in compressive strength. It was estimated that they contributed almost 50% of the total. The outcomes of this research have the potential to promote the reuse of PSPB in the building of green environments, hence boosting environmental protection and economic advantage.

## Introduction

Plastics are a type of man-made, synthetic material that is often employed in many applications. Because of their adaptability, plastics have seen dramatic growth in demand over the past few decades. About 300 million tonnes of plastic waste are created yearly in the globe, with 8 million of those tonnes ending up in the seas^1,2^. About 2.03 million metric tonnes of plastic waste were manufactured in Malaysia in 2018, as reported by the Malaysian Plastic Manufacturers Association (MPMA)^3^, with the majority going towards packaging needs and the remainder going to the electrical and electronic industries. Due to its limited biodegradability, the increased production of plastic waste has caused various environmental difficulties^4^. As more and more plastic waste is carelessly discarded into the environment, it contributes to pollution on land and sea and leaves behind harmful chemicals^5,6^. It is now generally accepted that plastic waste is a significant contributor to environmental contamination, particularly in marine and aquatic environments, with negative consequences for animals^7,8^. Due to its inertness and ability to pollute rivers, plastic waste has attracted a lot of attention in recent years^9,10^. According to research by Alabi et al.^6^ and Pinto Da Costa et al.^11^, the spread of plastic waste is correlated with human population growth, with higher numbers translating into more demand for plastic goods and, therefore, more pollution.

Numerous actions, including recycling and the prohibition of single-use plastic, have been made to lessen plastic waste buildup. Inefficient because of the time and effort required, less than 10% of plastic waste was recycled in the overall composition^12^. Due to its durability, plastic waste has been studied as a possible replacement for more traditional building materials^13^. In addition, plastic waste can improve the compressive strength, water absorption rate, and longevity of building materials. Plastic waste has been utilized in a variety of applications within the construction industry^14–17^. Research towards repurposing plastic waste as an alternative material in the building sector has been on the rise during the past decade. Many researchers have recently examined reusing plastic waste as a component of construction material^18–20^. While research has shown that plastic waste may be used in place of traditional materials, its practical use is limited. Since plastic waste reuse in building materials might lower compressive strength, there has been little consensus on the topic. Previously, researchers have found that plastic waste increases the product's compressive strength^21–23^. Therefore, it is important to examine the effects of incorporating plastic waste into the building material from the viewpoints of physical characteristics and compressive strength.

The typical building material for paver blocks is concrete, which consists of cement, sand, fine aggregates, and coarse aggregates. Because of the extensive use of materials and energy, particularly in the form of electricity, in the production of cement, the cement manufacturing sector is one of the largest contributors to greenhouse gas emissions. Cement is used as a binding agent. Emissions of greenhouse gases per tonne of cement produced are roughly estimated to be between 0.6 and 0.9 tonnes of CO_2_ equivalent^24^. The cement sector accounts for 5–6% of all worldwide greenhouse gas emissions, and cement output is rising at a pace of 8% per year for several countries^24–26^. The intergovernmental panel on climate change has urged the introduction of radically novel goods in place of cementitious ones^24,26^. Having been worth little more than $200 billion in 2020, the paver block market is forecast to reach $285.1 billion by 2025, a compound annual growth rate of 6.5%^27^.

The plastic-sand paver blocks (PSPB), which are made of solely plastic and sand, are one solution to the plastic waste issue and to the elimination of cement consumption in the construction industry. PSPB's mechanical qualities are affected by a number of variables, including the plastic used, the sand used, and the size of the sand^28–31^. The compressive strength of PSPB should be formulated. The employment of soft computing techniques is the superior choice for developing efficient mix design formulas and promoting the widespread application of hazardous materials in construction.

Recent studies on the extent of artificial intelligence (AI) techniques have aided in the development of consistent, dependable, and accurate models for structural engineering issues^32^. Various researchers have also used a variety of AI techniques to predict the properties of cement-stabilized soil^33–35^. AI approaches that use natural tools include the artificial neural network (ANN), genetic algorithm (GA), multi-expression programming (MEP), gradient boosting, and support vector regression (SVR)^36–46^. All aforementioned AI methods include "training" the solution using existing data. Artificial intelligence techniques such as support vector regression and artificial neural networks can recognize such intricate configurations and provide the resulting generalized pattern. Therefore, it serves a purpose in the broad field of engineering. These models are precise replicas; however, they do not provide an applicable empirical expression. Widespread implementation of the ANN model is slowed by its intricate design^47,48^. The ANN model was created by scientists to predict the punching shear strength of concrete. Overfitting occurs when the ANN's predicted values are compared to the values predicted by design codes. Because of their intricate makeup, they are difficult to manipulate^49,50^. Another issue that arises in such models is multicollinearity. The elastic modulus of recycled aggregate concrete and the compressive strength of silica fume concrete are both evaluated using the ANN method^51^. Due to the complexity of the planned connection, however, we were forced to depend only on a graphical user interface^51^.

Genetic programming (GP) differs from other methods of model development in that it does not rely on previously existing relations. The short programme is encrypted using fixed-length chromosomes in the more advanced version of GP known as gene expression programming (GEP)^52,53^. In addition, GEP provides a reliable empirical equation that has practical applications. As an alternative to other machine learning forecasting methods, it is being used in nearly all branches of civil engineering. The mechanical features of silica fume based light weight concrete, the fresh and hardened properties of self-compacting concrete, and the prediction of compressive strength in concrete made from rice husk ash are only a few examples^54–56^

A novel approach called multi-expression programming (MEP) has also been devised to address the aforementioned shortcomings of conventional machine learning methods. The MEP is special because it can store solutions to several equations (chromosomes) in a single piece of code. The optimal problem replica, based on the chosen chromosomes, is picked in the end^57^. MEP, an improved variant of GP, can compute a correct result even if the complexity of the objective is unknown, making it a competitive evolutionary algorithm^58^. MEP does not require the form of the ultimate Equation to be specified, unlike other ML methods. Mathematical contradictions are detected throughout MEP's development process and corrected in the final formulation. Decoding is also considerably easier in MEP than in other soft computing methods. Although MEP has several significant advantages over other evolutionary algorithms, it is not widely used in the civil engineering sector. The elastic modulus of both high- and normal-strength concrete has been predicted using MEP^40^. For the constrained concrete column, Arabshahi et al.^58^ suggested a design concept using aramid fibre reinforced polymers.

The compressive strength of PSPB has been the focus of a great deal of experimental study^29,59–62^. However, going with the experimental approach requires a lot of time and money. In order to link the compressive strength of PSPB to the mix percentage factors, it is preferable to construct a consistent, trustworthy, and precise Equation. The literature shows that there are no MEP-based empirical equations or GEP-based empirical equations for approximating the compressive strength of PSPB. These models, however, are constructed using the relevant experimental results. This work employs the GEP and MEP machine learning approaches to fill this knowledge gap and produce a precise expression for the approximate future compressive strength of PSPB. Consistency, dependability, and correctness of the established models for unknown data were ensured by a thorough and vast database. A simplified equation for both GEP and MEP was developed for the PSPB. An intensive statistical, k-fold, and sensitivity analysis was conducted to assess the generalizability of the built models. Finally, linear and non-linear regression expressions were used to compare the GEP model to the MEP model.

## Research methodology

**Figure 1:**
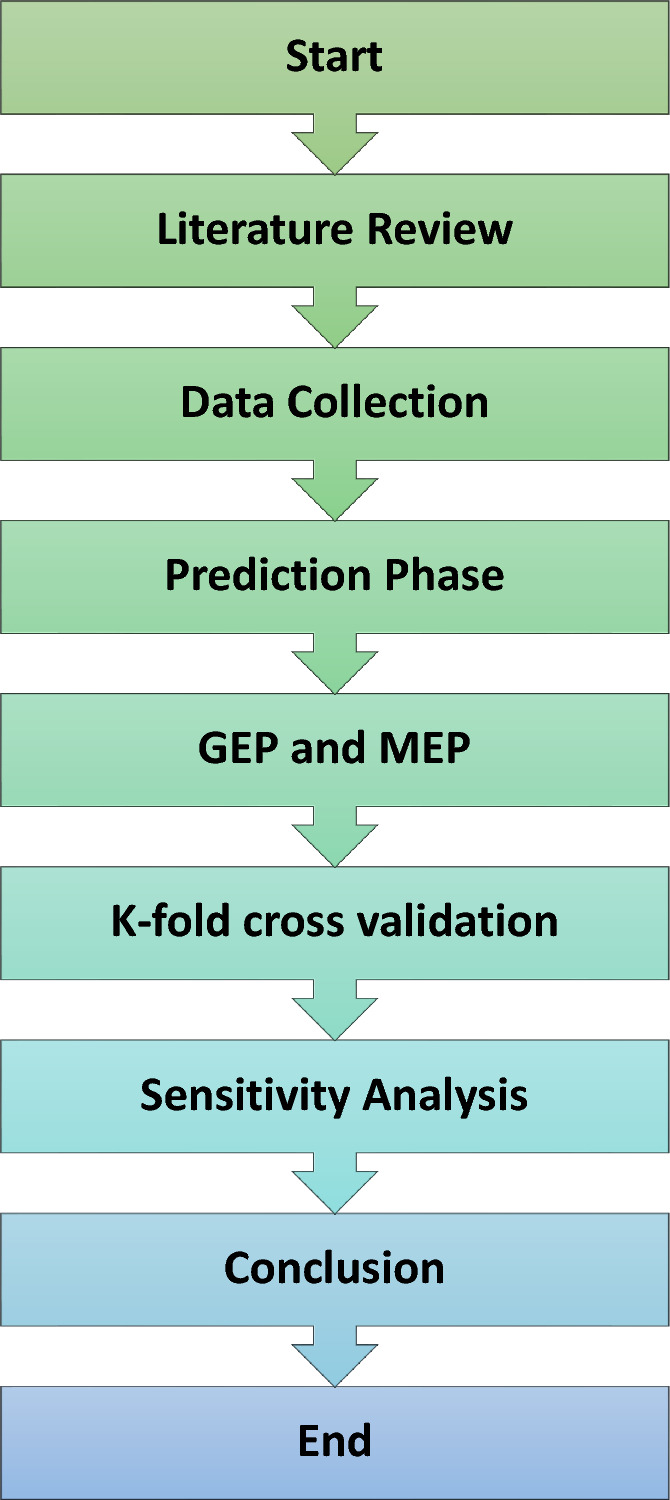
Flow chart of the research.

This section discusses the process followed when developing a GEP and MEP-based Equation to determine the compressive strength of PSPB. Following a brief summary of GEP and MEP, the study's methodology will be discussed. The methodology flow chart is shown in Fig. 1.

### Gene expression programming

To address the need for a different approach to fixed-length binary strings (used in genetic algorithms), Koza presented a GP technique^63^. The GP methodology defines five main parameters: the gathering of terminals, the set of primitive functions, the level of fitness evaluation, the control variables, and the conditions for termination accompanied by the outcomes classification method^63^. GP is a flexible programming method because it may be used to induce non-linear structures that resemble parse trees. It presupposes any non-linearity from the outset, given the data. Similar non-linearities have been employed in the past^63,64^. The inability to account for a person's unique genome is a major shortcoming of GP. The genotype and phenotype in GP have the same non-linear structure. This reduces the likelihood that naive or unsophisticated language may result. To address the shortcomings of the GP approach, Ferreira proposes the GEP method^63^. The fact that just the genome is passed down from one generation to the next is a major change throughout GEP. The formation of entities by an individual chromosome containing several genes is another notable feature^65^. Each gene in GEP is represented by a collection of terminal constants and a fitted length parameter, and the functions are the arithmetic operations. In addition, the relationship between the related function and the chromosomal symbol is stabilized in genetic code operators. Data required to build an empirical model is written to chromosomes, and a new programme called karva is created to deduce their meaning.

**Figure 2:**
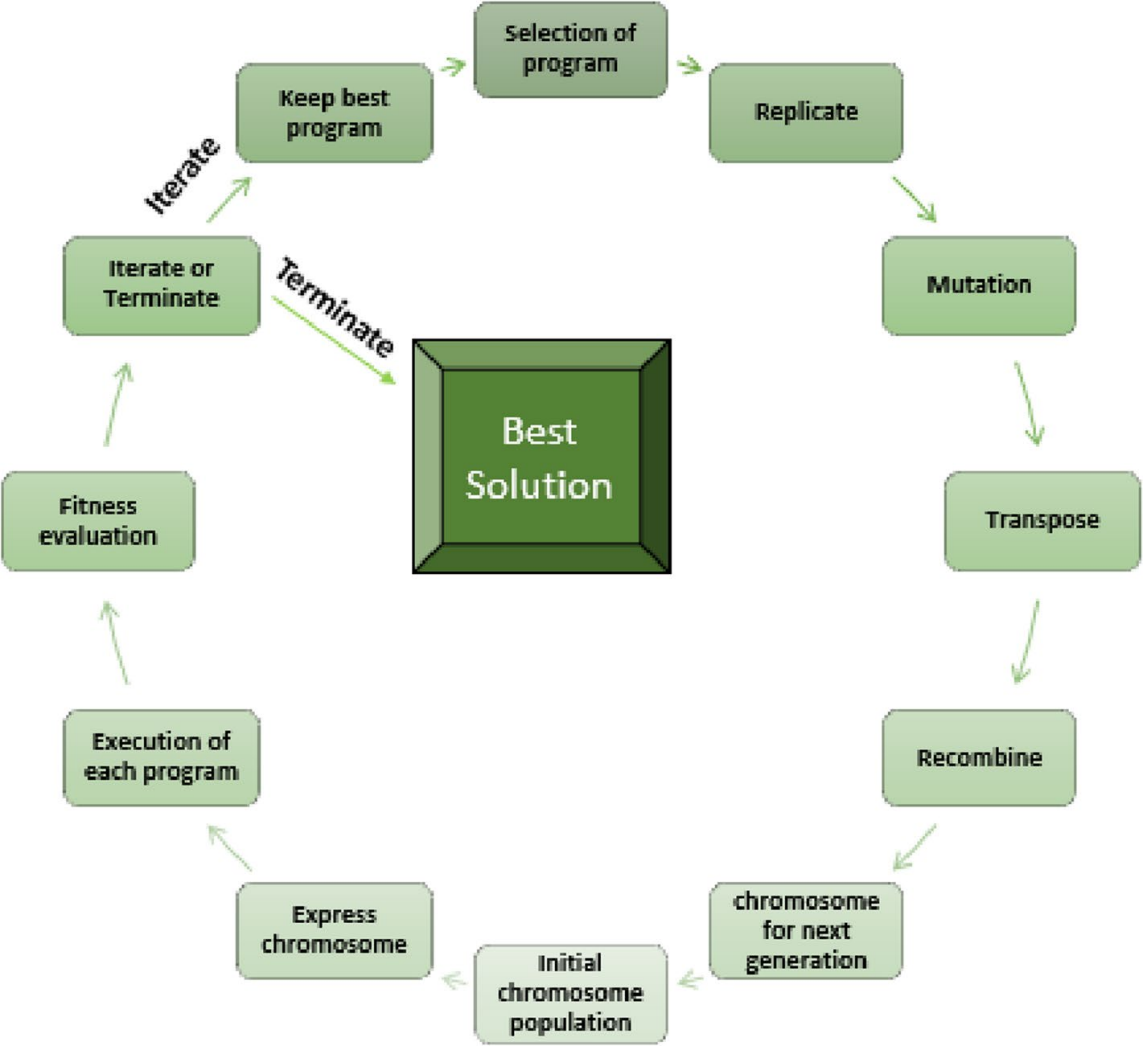
Steps involved in GEP.

The steps involved in GEP are depicted in Fig. 2. Starting with randomly generated chromosomes of the same size for each individual, the approach then converts them into expression trees (ET) and calculates an estimate of fitness for every single individual. Replication with fresh new individuals continues for several creations until desirable outcomes are reached. Populations may be changed by employing genetic operations like crossover, reproduction, and mutation.

### Multi expression programming

**Figure 3:**
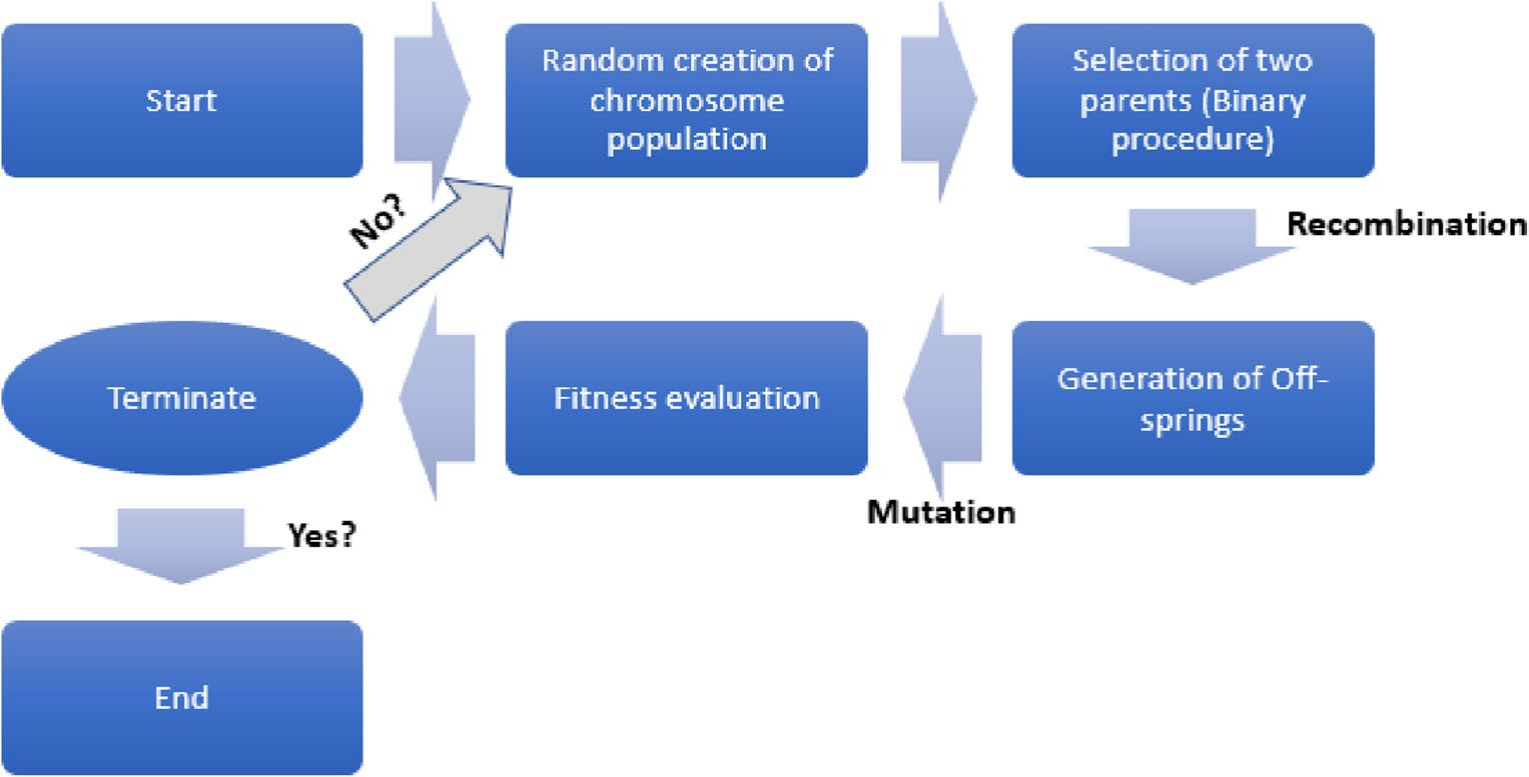
Steps involved in MEP.

The MEP is a thorough, proven linear-based GP method that uses linear chromosomes to encode data. The working mechanism of the MEP is similar to that of the GEP. The ability to encrypt many software packages (solutions) onto a single chromosome^66^ is a crucial part of MEP, which is a unique subset of the GP approach. Then, the best chromosome is selected by assessing fitness values to generate the final product. According to Oltean and Grosan^67^, a binary environment that splits into two offspring would inevitably choose two parents. The procedure is repeated until the optimal programme is found, at which point the criteria are stopped. This is the site where future generations begin to change. The MEP model, like the GEP model, allows for parameter fitting. The key variables that govern multi-expression programming are the number of code lengths, subpopulations, crossover probability, subpopulation size, and set of functions^68^. When the population size is the total number of programmes, the computation and time required to calculate are compounded as the number of subpopulations increases. In addition, the length of the code has a major impact on the size of the resulting mathematical expressions. Figure 3 shows the steps involved in the MEP technique.

### Comparison of GEP and MEP

Historical data sets are generally utilized throughout the assessment and modeling phases for every of the aforementioned genetic programming approaches^69,70^. It is often believed that the GEP and MEP methods, in particular, are the most prominent linear GP methodologies that properly assess the compressive strength of the concrete composite. When compared to that of the MEP, the operating system of the GEP possesses a higher degree of complexity^68^. In contrast to the GEP, the noncoding parts of the MEP can be located wherever on the chromosome. Additionally, connections to function attributes are clearly documented in the MEP method^57,67^. Because of these changes, the MEP format is more suited for the reuse of code (despite the fact that it is less condensed than GEP). In addition, it is stated that it contains the head and tail of a typical GEP chromosome. The head and tail of a typical GEP chromosome both include symbols that successfully represent syntactically logical computer programmes, which is further evidence that the GEP is far more effective. As a direct consequence of this, more study is needed to evaluate the efficacy and the applicability of both GP approaches to a particular engineering problem. GEP and MEP are both used to discover answers to optimization issues; however, whereas MEP focuses primarily on identifying a single equation that can be utilized to solve a problem, GEP is more focused on modeling and approximating data. GEP is employed to identify solutions to optimization problems.

The benefits of MEP are as follows^71,72^; MEP use many expression sets rather than a single expression. As a result, the material strength prediction model may be broken down into its constituent parts, or phases, which can then be more easily understood and analysed. The computing time required to evolve the model and estimate concrete strength may be drastically decreased by evaluating the many expression sets used in MEP in parallel. This feature of parallel processing becomes very useful when dealing with enormous datasets. The concept of epistasis, which describes how different genes or expressions influence one another, is incorporated into MEP. As a result of epistasis's incorporation, MEP is able to account for the complex interplay between the different variables that affect the durability of concrete.

### Criteria for assessing models

When evaluating a model's performance on a training or testing set, statistical errors such as mean absolute error root (MAE), root mean square error (RMSE), R-square value (R^2^), and normalized root mean squared error (NRMSE), and were used. A model's predictive ability is quantified by its R^2^ (also referred to as the determination coefficient)^73,74^. Improvements in artificial intelligence (AI) modeling approaches have allowed for more precise predictions of concrete's mechanical properties. In this research, the GEP and MEP models are statistically compared by the calculation of error criteria. There are a lot of measures that might help explain why the model is inaccurate. The coefficient of determination may be used to verify the reliability and validity of the model. Models with R^2^ values that are more than 0.50 produce disappointing results, whereas models with R^2^ values that fall within the range of 0.65 and 0.75 produce encouraging results. Equation (1) may be used to determine R^2^. Both the input and the output of MAE use the same units. It is possible for a model with an MAE within a certain range to make serious errors on occasion. In order to determine MAE, we use Eq. (2). The RMSE is the average squared error in estimations and measurements. Error squared is calculated by summing the error squares. This new approach pays greater weight to extreme cases than did earlier calculations, producing large squared differences in some cases but smaller ones in others. As the RMSE number drops, the model's ability to accurately forecast new data improves. The RMSE is computed using Eq. (3). The RMSE is helpful for comparing models of varying complexity. An alternative to the RMSE that accounts for the variable's observed spread is the NRMSE. So, the NRMSE can be thought of as a fraction of the total range that the model can usually resolve. Using Eq. (4), we can calculate the NRMSE. Recently, various analyst worked on different materials applications like civil engineering and sustainability^75–77^, prediction of mine water in flow and cement based materials^78–80^, structure engineering applications^81–83^, reinforced reservoir, thermal evolution of chemical structure and concrete beam^84–86^, fiber reinforced soil^87^, stress relaxation behavior^88^ and embankment and foundation for ballast less high speed railway^89^.

1 $${R}^{2}= 1-\frac{\sum_{j=1}^{m}{({p}_{j}-{t}_{j})}^{2}}{\sum_{j=1}^{m}({t}_{j}-\overline{t })}$$

2 $$MAE= \frac{{\sum }_{j=1}^{m}\left|{t}_{j}-{p}_{j}\right|}{n}$$

3 $$RMSE= \sqrt[ ]{\frac{{\sum }_{j=1}^{m}{\left({t}_{j}-{p}_{j}\right)}^{2}}{n}}$$

4 $$NRMSE= \frac{RMSE}{\overline{t} }$$

## Data collection

**Figure 4:**
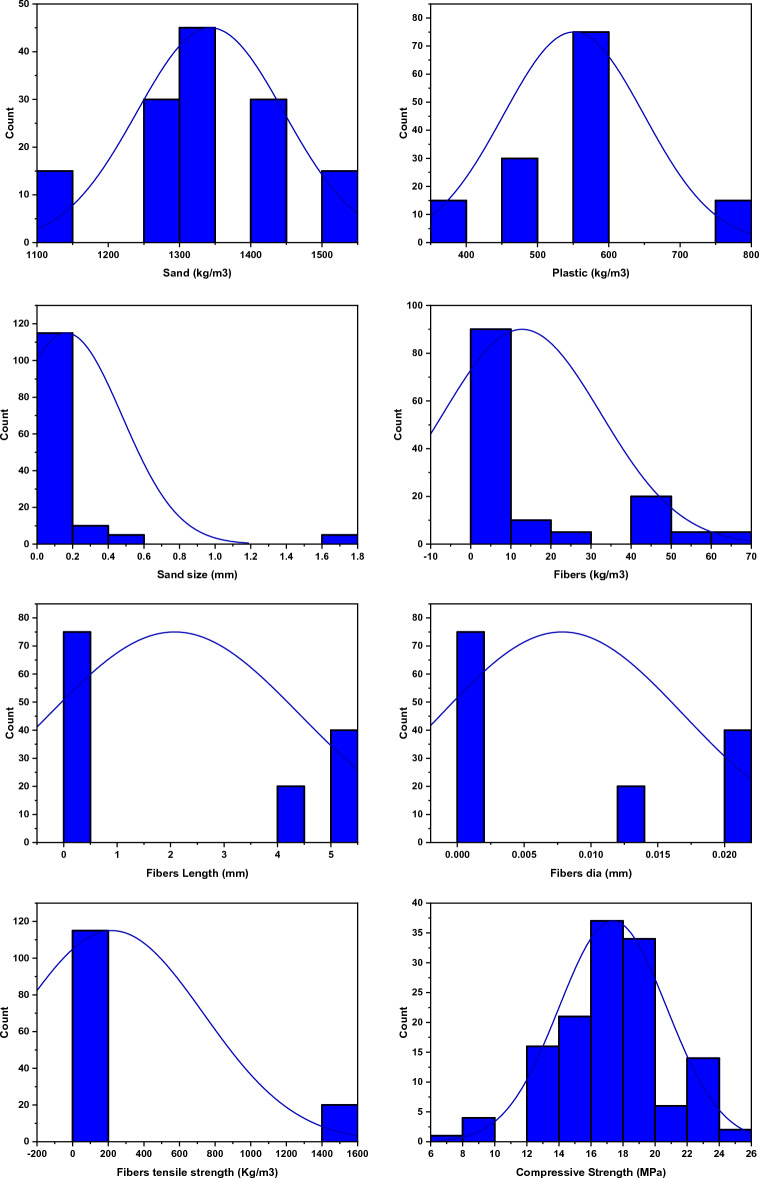
Frequency distribution of the data utilized in the creation of the models (**a**) sand, (**b**) plastic, (**c**) sand size, (**d**) fiber percentage, (**e**) fiber length, (**f**) fiber diameter, (**g**) fiber tensile strength, (**h**) compressive strength.

Our study relied on actual experimental testing that was performed in a laboratory facility. The PSPB was manufactured with a wide range of plastic-to-sand ratios, sand sizes, various fibre percentages, and fibre lengths. The data included in the models were derived from experiments done in the past^90,91^. The compressive strength has been calculated through laboratory testing of 135 samples. The materials involved in the developing of this PSPB were plastic, sand and fibers (basalt fibers and coconut fibers). Table 1 displays the input and output parameters considered in this analysis. The studies with the most promising outcomes are selected for further analysis. Seven input parameters (plastic, sand size, length of fiber, sand, percentage of fiber, diameter of fiber and tensile strength of fiber) were chosen from the literature, while all other variables were held constant, and modelling was performed on this data set. Similar approaches were reported in the prior literature, wherein the other factors, such as curing regimens, method of preparation, physical and mechanical properties of raw materials, and environmental condition, were held constant^92–95^. Figure 4 and Table 2 present the model's frequency distribution and generic data descriptions, respectively. Distribution plays a role in the effectiveness of any model^96^. It should be noted that multiple tests were performed to ascertain the database's validity and accuracy. The data with the highest error rates were disregarded, while those with the lowest error rates were chosen for the model prediction^97^. Models are tested and trained with the use of the GEP and MEP methods in this study. The models were trained on 80% of the data and then tested on the remaining 20%. The results of this testing provide a precise complement to those of previous experimental testing conducted on a variety of models. Since the research employs several models, the correctness of each model has previously been confirmed and evaluated using testing data. Genetic evolution was used to train the model, while testing data was used to verify the accuracy of the embedded model^39,98^.

**Table 1:** Input and Output parameters involved in the modeling.

Parameters	Abbreviation
Input variables	
Plastic (kg/m^3^)	P
Sand (kg/m^3^)	S
Sand size (mm)	SS
Fiber length (mm)	FbL
Fiber percentage (kg/m^3^)	Fb
Fiber diameter (mm)	FbD
Fiber tensile strength (MPa)	FbT
Output variable	
Compressive strength (MPa)	fc′

**Table 2:** Descriptions of variables used in modeling.

	Total	Mean	Standard deviation	Sum	Minimum	Median	Maximum
Sand (kg/m^3^)	135	1341.57432	415.49081	3,144,972.181	1144.2	1330.8953	1525.6
Plastic (kg/m^3^)	135	552.75415	397.33428	1,295,788.384	381.4	572.1	762.8
Sand size (mm)	135	0.14882	1.2057	348.87025	0.075	0.075	1.69
Fibers (kg/m^3^)	135	12.67153	78.61875	29,705.11588	0	0	66.745
Fibers Length (mm)	135	2.14517	9.77551	5028.79	0	0	5
Fibers dia (mm)	135	0.00808	0.03771	18.95173	0	0	0.02
Fibers tensile strength (MPa)	135	245.80875	2242.74152	576,234.7	0	0	1450
Compressive Strength (MPa)	135	18.01271	13.14766	42,226.1106	7.2	17.9	24

## Results and discussion

### K-fold cross-validation

**Figure 5:**
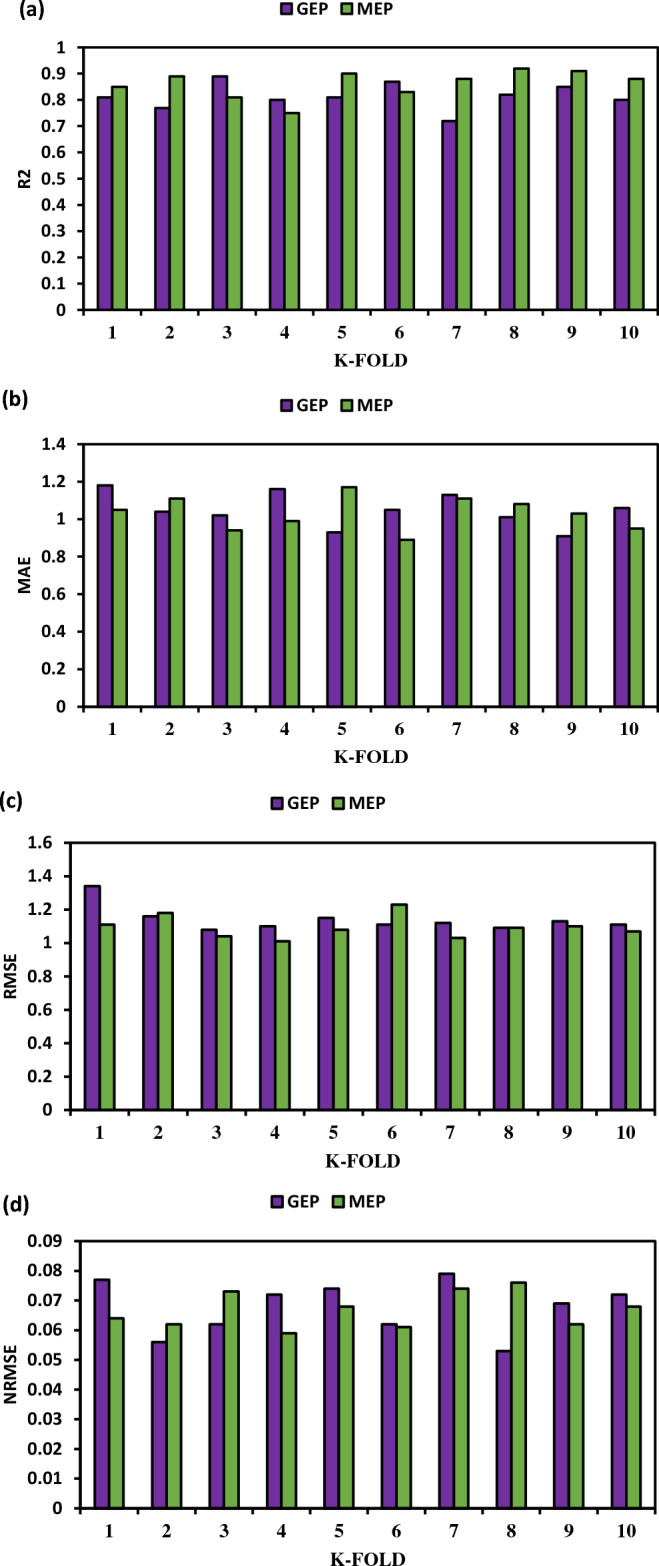
K-fold cross-validation of PSPB for (**a**)R^2^, (**b**)MAE, (**c**)RMSE, (**d**)NRMSE.

Researchers from a range of domains have hypothesized that the ratio of data indicates that the overall quantity of inputs has a significant role in the effectiveness of the suggested model^96,99^. For the best model models^99^, the proportion should be more than 5 so that data points may be tested for their ability to determine the link between the chosen variables. The present study predicts the compressive strength of the PSPB using seven inputs, and the resultant proportion of 19.2 meets the requirements set out by the researchers. The findings of k-fold cross-validation using GEP and MEP yielded insightful information on the effectiveness of these methods. Maximum R^2^ values for GEP were 0.89, minimum R^2^ values for GEP were 0.72, and the average R^2^ value for GEP was 0.81. However, MEP demonstrated somewhat better performance, with an R^2^ range of 0.92 to 0.75 and an average of 0.86. These results suggest that both GEP and MEP are adequately fitting the data, with MEP providing a slightly superior overall fit, as shown in Table 3. The MAE results were also calculated, and the results for GEP ranged from 1.18 to 1.91, with 1.04 being the average value. Similar encouraging outcomes were seen with MEP, which had an MAE range from 1.17 to 0.89 to 1.02. By showing the average absolute difference between the anticipated and actual values, these numbers shed light on the reliability of the models. In terms of MAE, both GEP and MEP performed well, indicating that they can generate reliable predictions. In terms of the RMSE statistic, GEP ranged from 1.34 to 1.08, with an average of 1.03. In contrast, MEP's RMSE ranged from 1.23 to 1.01, with an average of 1.09. Finally, GEP was measured using the NRMSE metric, which gave an extreme range of values (0.079 to 0.053) with an average of 0.067. By dividing the RMSE by the target variable's range, the NRMSE measure standardizes the RMSE. NRMSE values for MEP ranged from 0.076 to 0.059, with an average of 0.066. Figure 5 shows the result of the k-fold cross-validation for both GEP and MEP. In terms of prediction accuracy and variability with respect to the target variable, the NRMSE values that are closer to zero are indicative of higher performance. In conclusion, when tested using k-fold cross-validation, both GEP and MEP showed signs of being highly effective. Overall, MEP performed better than GEP, with lower MAE, RMSE, and NRMSE values and higher R^2^ values. These results demonstrate the promise of both GEP and MEP, while the overall greater performance of MEP suggests it may be a better fit for this dataset. Similar results were also reported previously showing MEP performed better than GEP^100,101^ .

**Table 3:** K-fold values of PSPB for MEP and GEP.

K-fold	GEP	MEP	GEP	MEP	GEP	MEP	GEP	MEP
	R^2^	R^2^	MAE	MAE	RMSE	RMSE	NRMSE	NRMSE
1	0.81	0.85	1.18	1.05	1.34	1.11	0.077	0.064
2	0.77	0.89	1.04	1.11	1.16	1.18	0.056	0.062
3	0.89	0.81	1.02	0.94	1.08	1.04	0.062	0.073
4	0.8	0.75	1.16	0.99	1.1	1.01	0.072	0.059
5	0.81	0.9	0.93	1.17	1.15	1.08	0.074	0.068
6	0.87	0.83	1.05	0.89	1.11	1.23	0.062	0.061
7	0.72	0.88	1.13	1.11	1.12	1.03	0.079	0.074
8	0.82	0.92	1.01	1.08	1.09	1.09	0.053	0.076
9	0.85	0.91	0.91	1.03	1.13	1.1	0.069	0.062
10	0.8	0.88	1.06	0.95	1.11	1.07	0.072	0.068

In addition, statistical error evaluations were carried out in order to evaluate the effectiveness of the model, as shown in Table 4. The models were dependable and accurate in their predictions of the compressive strength of PSPB, as shown by the RMSE, MAE, and NRMSE values. In addition, the statistical error check reveals that MEP is exhibiting better predictive outcomes than GEP by displaying lower values of MAE, RMSE, and NRMSE.

**Table 4:** Statistical checks.

	MAE	RMSE	NRMSE
MAE	1.007	1.174	0.069
RMSE	0.983	1.158	0.066

### Developing the PSPB empirical equation using GEP

**Figure 6:**
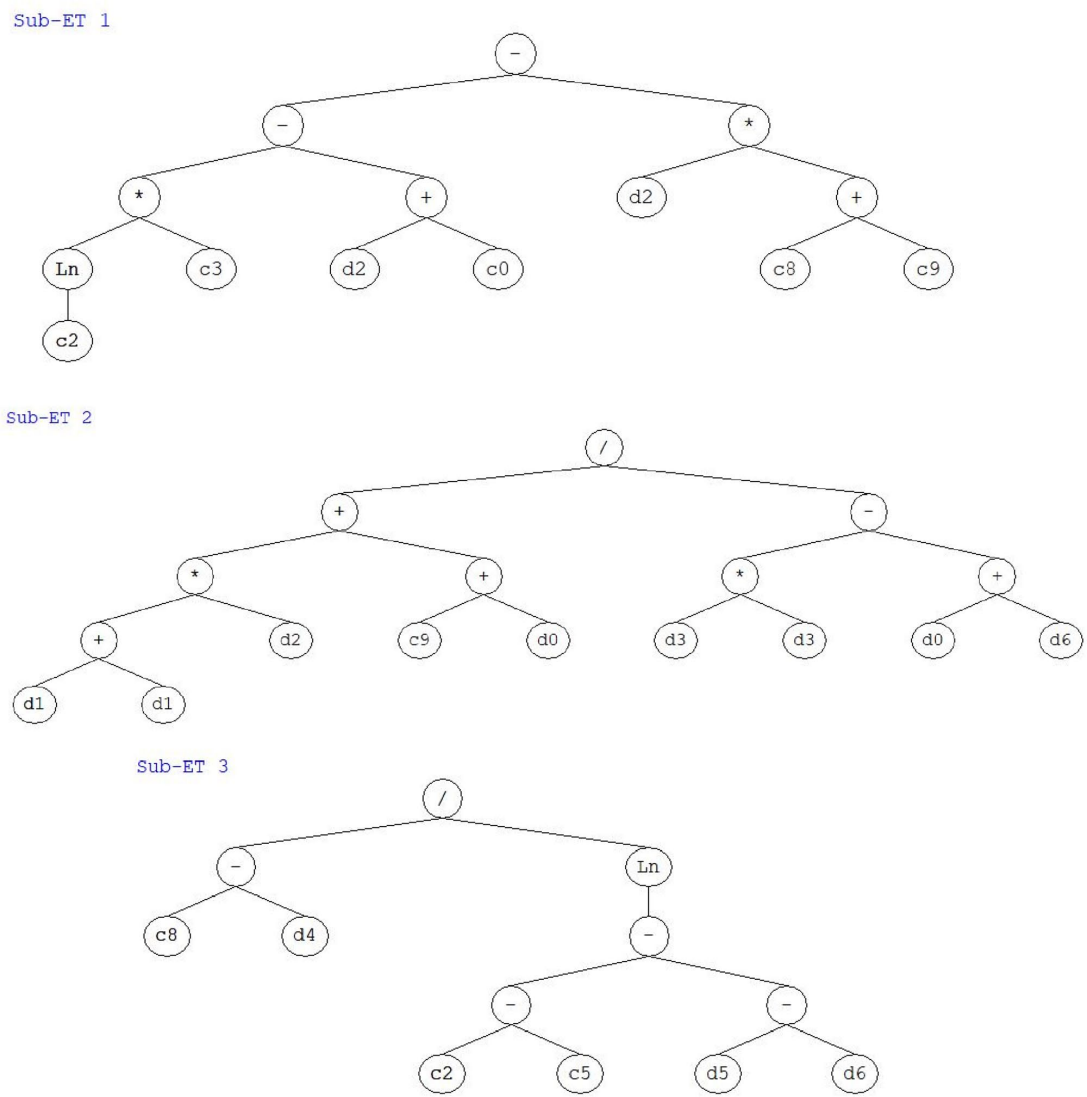
Expression trees for the PSPB to forecast compressive strength.

GEP was used to develop an empirical equation for PSPB's compressive strength. By fusing genetic programming with a classic genetic algorithm, GEP creates a potent evolutionary algorithm. The objective was to formulate a formula that, given a number of input parameters, reliably predicted the compressive strength of the paver blocks. To begin, seven input variables were defined, each of which was selected for its potential impact on compressive strength. Then, the input variables and the five arithmetic operators were selected to form the terminal set. The GEP method resulted in the creation of expression trees (ETs), which were constructed using these terminal symbols as their basis and comprised of the five basic arithmetic operations, i.e., −, + , x, ÷ , and Ln. The PSPB GEP model's ETs are depicted in Fig. 6. The GEP method iterated and refined the expression trees to arrive at the best possible empirical Equation for the compressive strength over the course of several generations. Each ET was given a fitness score, with the best individuals being chosen, mutated, crossed across, and tested again until an excellent response was found.

Following the identification of the three sub-expression trees (sub-ETs), the final empirical Equation for the compressive strength of the plastic sand paver blocks (PSPB) was formed by combining the results of these equations. This Equation depicts the link between the input factors and the compressive strength, as shown in Eq. (5). It provides helpful insights as well as a prediction tool that can be used in the process of designing and producing paver blocks that are durable.

5 $$CS = A+B+C$$

where,

$$A =Ln(7.09) \times (9.48) - (SS - 3.13) - SS \times (8.46)$$

$$B =\frac{((2 \times P) \times SS)+(-9.82+S)}{(2 \times Fb) - (S+FbT)}$$

$$C =\frac{-6.16 - FbL}{Ln(14.13 - (FbD - FbT))}$$

### Performance of the GEP model

**Figure 7:**
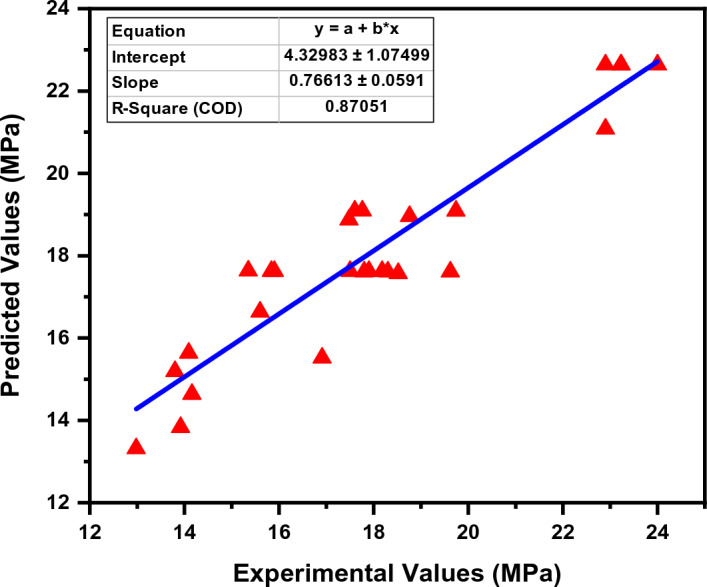
GEP actual and predicted values.

**Figure 8:**
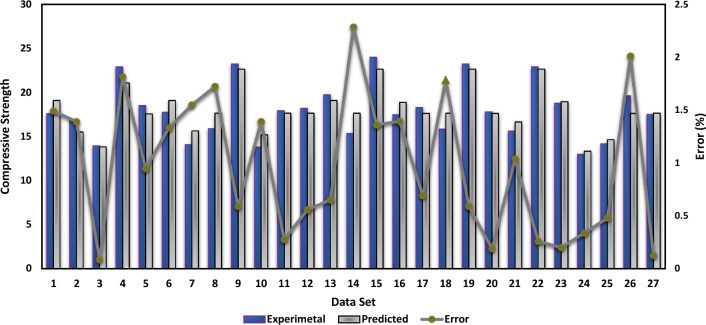
GEP error distribution.

Through the use of GEP, the compressive strength of the PSPB was analyzed to determine how effectively they performed. The evaluation came up with an R^2^ value of 0.87, which indicates a relatively good match between the empirical Equation established using GEP and the actual compressive strength data, as shown in Fig. 7. In order to determine the error between the experimental and predicted values, the error was analyzed as well. The compressive strength that was predicted was significantly different from the actual values by 2.28 MPa, which was the amount of inaccuracy that was observed to be the greatest. On the other hand, the error that was recorded as being the lowest was 0.08 MPa, which indicates that the real compressive strength was approximated quite closely. An overall measure of the difference between the anticipated values and the actual values was found to be 0.98 MPa, which was determined to be the average value of the error. Figure 8 shows the error distribution of the actual and predicted dataset. Additional research was carried out in order to classify the mistakes according to the extent of their occurrence. It was found that 29.6% of the errors were less than 0.5 MPa, which indicates a good level of accuracy in forecasting compressive strength within a restricted range. A reasonable degree of accuracy was indicated by the fact that 48.1% of the total errors were within the range of 0.5 MPa to 1.5 MPa. On the other hand, 22.3% of the total was made up of mistakes that were more than 1.5 MPa, which indicates that the prediction model needs additional improvements. These findings provide evidence that GEP is an excellent method for developing an empirical equation for determining the compressive strength of PSPB.

### Developing the PSPB equation using MEP

In this part, multinomial expression models are developed in order to make a prediction about the compressive strength of PSPB based on seven different parameters that were input. In addition, Eq. (6) contains empirical equations obtained from ETs for the output of PSPB that was used to identify the compressive strength result. These empirical equations can be utilized to estimate the compressive strength result. In addition, the ETs are made up of the same five arithmetic operators as before, i.e., −, + , ×, ÷ , and Ln.

6 $$CS =\frac{SS+S+Fb}{Fb+SS+Ln(P)+2 \times Ln(\frac{P+Fb}{SS}) - S}+ Ln(\frac{P+Fb}{FbD}) \times SS+2 \times Ln(\frac{P+FbT}{FbD}) -\frac{Fb}{SS+2 \times (FbL)}+\frac{SS+2 \times (FbL)}{Ln(\frac{P+Fb}{SS}) - Fb}$$

### Performance of MEP model

**Figure 9:**
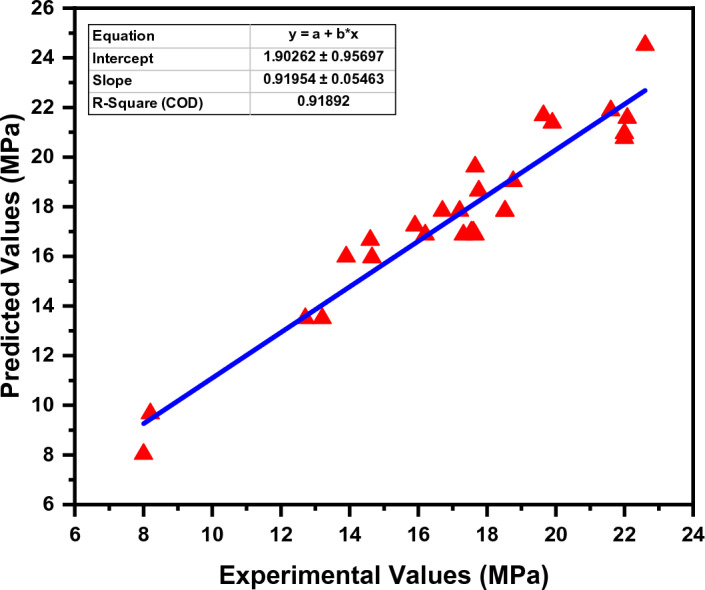
MEP experimental and predicted values.

**Figure 10:**
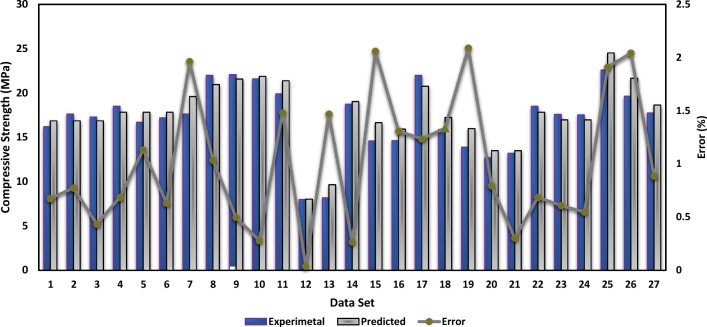
Error distribution of the MEP.

The effectiveness of PSPB in terms of compressive strength was investigated by employing a technique known as MEP. The assessment procedure produced an R^2^ value of 0.91, which indicates a significant connection between the empirical Equation derived by MEP and the actual compressive strength values. Figure 9 depicts the actual and predicted values of the MEP model. In order to determine the difference between the actual and predicted values, the errors were analyzed as well. The error distribution of the MEP model is shown in Fig. 10. The projected compressive strength was significantly different from the actual readings by a total of 2.09 MPa, which was the biggest inaccuracy that was recorded during the experiment. On the other hand, the error that was reported as being the smallest was 0.03 MPa, which indicates that the real compressive strength was approximated quite closely. An overall measure of the divergence between the projected and actual values was determined to be 1 MPa, which was found to be the average amount of inaccuracy that was detected. Additional research was carried out in order to classify the mistakes according to the extent of their occurrence. It was found that 22.2% of the errors were less than 0.5 MPa, which indicates a high degree of accuracy in forecasting compressive strength within a restricted range. This finding was made possible by the fact that the range of the data was narrow. Errors that fell between the range of 0.5 MPa to 1.5 MPa made up 59.3% of the total, which indicates that a sizeable number of accurate forecasts were within the moderate range. On the other hand, errors bigger than 1.5 MPa accounted for 18.5% of the total, which indicates that the predictive model has less room for error variations than GEP. These findings provide evidence that Multi Expression Programming is a viable method for developing an empirical equation for determining the compressive strength of plastic sand paver blocks. The accuracy of the model appears to be promising, given that it has a high R^2^ value and the bulk of its predictions are within error limits that are acceptable.

### Sensitivity analysis

Sensitivity analysis is a useful method for evaluating the effect of varying input variables on the predicted outcome of a model. This technique is essential for comprehending the model's behavior and dependability^102^. To commence the sensitivity analysis, it is necessary to precisely define the issue while determining the input variables that influence the model's output. After identifying the variables, the next stage was to determine the range of possible values for each input variable. This range should include reasonable and significant values for the parameters under consideration. Sensitivity analysis allows us to assess the relative relevance and impact of each input variable on the model's output by examining various values within the defined ranges. This process aids in determining which variables have the greatest influence on the predictions and facilitates the making of well-informed decisions based on the behavior of the model. In the instance of PSPBs, a sensitivity analysis was carried out to determine the impact of a number of different elements on their performance regarding compressive strength. Recently, various analyst worked on different materials applications like civil engineering and sustainability^95–97^, prediction of mine water in flow and cement based materials^98–100^, structure engineering applications^75,101,102^, reinforced reservoir, thermal evolution of chemical structure and concrete beam^76–78^, fiber reinforced soil^79^, stress relaxation behavior^80^ and embankment and foundation for ballast less high speed railway^81^.

Equations (7) and (8) were used in the process of carrying out the sensitivity analysis.

7 $${N}_{i}={f}_{max}{(x}_{i})- {f}_{min}{(x}_{i})$$

8 $$SA=\frac{{N}_{i}}{\sum_{n}^{j=1}{N}_{j}}$$

where, ${f}_{min}{(x}_{i})$ = forecasting model (minimum outcome), ${f}_{max}{(x}_{i})$ = forecasting model (maximum outcome), i = representing the range of inputs while keeping all other factors fixed.

**Figure 11:**
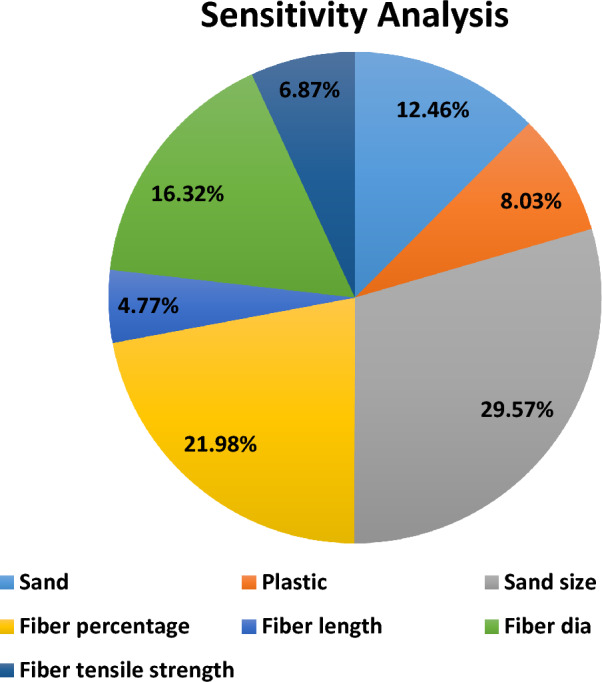
Sensitivity analysis of the PSPB.

The findings presented the percentage contribution that could be attributed to each element, so giving light to the relative importance of the variables. It was discovered that the size of the sand had the biggest contribution of around 29.57% among the components that were evaluated, demonstrating the enormous effect that it has on the performance of the blocks. It was discovered that the proportion of fibres that were included in the blocks had a significant influence, with a contribution that was around 21.98% of the total. Other parameters, such as fibre length (4.77%), fibre diameter (16.32%), and fibre tensile strength (6.87%), provided significant contributions to the compressive strength of the plastic sand paver blocks as well. These findings give useful insights for optimizing the manufacture and composition of plastic sand paver blocks, which are currently in use. Figure 11 shows that all of the variables have an important role in predicting PSPB's compressive strength.

## Conclusion

No research has been done on the PSPB to generate the empirical Equation utilizing GEP and MEP methods. To address this information gap and generate an accurate expression for anticipating the compressive strength of PSPB, the current work utilizes the GEP and MEP machine learning methodologies. The constructed models' generalizability was evaluated using extensive statistical, k-fold, and sensitivity analyses. The GEP and MEP models were compared using linear and non-linear regression expressions. The following are some of the particular findings of this study.

- The compressive strength R^2^ values of 0.87 for GEP and 0.91 for MEP indicate a relatively strong correlation between predicted and actual values. In terms of R^2^, MEP outperformed GEP, indicating a superior fit to the data.
- MEP developed a unique mathematical equation to predict compressive strength, indicating that it was more effective than GEP at capturing the underlying patterns and relationships in the data.
- The statistical error measures (MAE, RMSE, and NRMSE) were lower for MEP (i.e., 0.983, 1.158, and 0.066) than they were for GEP (i.e., 1.007, 1.174, and 0.069), indicating greater precision in predicting compressive strength.
- The results of k-fold cross-validation consistently demonstrated that MEP outperformed GEP in terms of compressive strength prediction. This demonstrates the model's robustness and generalizability.
- According to a sensitivity analysis, sand size and fibre percentage had roughly half the impact on compressive strength as the other five input parameters. This emphasizes the significance of regulating and optimizing these variables to increase PSPB's compressive strength.

The created models might be utilised to determine the compressive strength of PSPB for a variety of input parameter values, saving time and money on future trials. This study was limited to using seven fundamental variables (P, S, SS, FbL, Fb, FbD and FbT) for developing prediction models. However, other factors like curing regime, method of preparation, environment condition also impact the strength of a material. Therefore, further studies are required to generate a more comprehensive database including all possible influential parameters to develop models for strength evaluation of the materials.

## Data Availability

The datasets used and/or analysed during the current study available from the corresponding author on reasonable request.
